# Comedo-DCIS is a precursor lesion for basal-like breast carcinoma: identification of a novel p63/Her2/neu expressing subgroup

**DOI:** 10.18632/oncotarget.818

**Published:** 2013-02-24

**Authors:** Malathy P.V. Shekhar, Ikuko Kato, Pratima Nangia-Makker, Larry Tait

**Affiliations:** ^1^ Department of Oncology, Wayne State University, Detroit, MI, U.S.A.; ^2^ Karmanos Cancer Institute, Detroit, MI, U.S.A.

**Keywords:** breast cancer, p63, Her2/neu, cytokeratin, EGFR

## Abstract

Basal breast cancer comprises ~15% of invasive ductal breast cancers, and presents as high-grade lesions with aggressive clinical behavior. Basal breast carcinomas express p63 and cytokeratin 5 (CK5) antigens characteristic of the myoepithelial lineage, and typically lack Her2/neu and hormone receptor expression. However, there is limited data about the precursor lesions from which they emerge. Here we wished to determine whether comedo-ductal carcinoma in situ (comedo-DCIS), a high-risk in situ breast lesion, serve as precursors for basal-like breast cancer. To determine this link, p63, CK5, Her2/neu, epidermal growth factor receptor (EGFR), estrogen receptor (ER) and progesterone receptor (PgR) expression were analyzed by immunohistochemistry in 17 clinical comedo- and 12 noncomedo-DCIS cases, and in tumors derived from unfractionated and CK5-overexpressing subpopulation (MCF10DCIS.com-CK5^*high*^) of MCF10DCIS.com cells, a model representative of clinical comedo-DCIS. p63 and Her2/neu coexpression was analyzed by immunofluorescence double labeling. A novel p63/CK5/Her2/neu expressing subpopulation of cells that are ER^*−*^/PgR^*−*^/EGFR^*−*^ were identified in the myoepithelial and luminal areas of clinical comedo-DCIS and tumors derived from unfractionated MCF10DCIS.com and MCF10DCIS.com-CK5^*high*^ cells. These data suggest that p63 and Her2/neu expressors may share a common precursor intermediate. P63, but not Her2/neu, expression was significantly associated (*P* = 0.038) with microinvasion/recurrence of clinical comedo-DCIS, and simultaneous expression of p63 and Her2/neu was marginally associated (*P* = 0.067) with comedo-DCIS. These data suggest that p63/Her2/neu expressing precursor intermediate in comedo-DCIS may provide a cellular basis for emergence of p63+/Her2/neu- or p63+/Her2/neu+ basal-like breast cancer, and that p63/Her2/neu coexpression may serve as biomarkers for identification of this subgroup of basal-like breast cancers.

## INTRODUCTION

Clinical breast cancer consists of a heterogeneous group of tumors that are only partially distinguishable by morphological presentation. Molecular profiling studies have helped to further resolve the heterogeneity of tumors that are not discernible by morphological evaluation. Five distinct subtypes have been identified by molecular profiling: basal (ER^−^/PgR^−^/Her2^−^/CK5+ that are triple negative for estrogen receptor (ER), progesterone receptor (PgR) and Her2/neu and positive for cytokeratin 5 (CK5), luminal A (ER+/Her2^−^), luminal B (ER+/Her2+), Her2-overexpressing, and normal-like [1,2]. The basal subtype of breast cancer represents approximately ~15% of invasive ductal breast cancers. Histologically, basal breast cancers present as high grade lesions with areas of necrosis, lymphocytic infiltration, poor nuclear grade and high proliferation rate [3]. Epidermal growth factor receptor (EGFR) is overexpressed in ~38% of basal carcinomas [4], however, clinical trials with EGFR tyrosine kinase inhibitors and Cetuximab have been largely disappointing [5]. Basal or basal-like breast carcinomas express p63, CK5, CK6, CK14 and CK17 proteins that are typically expressed in myoepithelial cells of the mammary epithelium [3, 6-11], while luminal tumors rarely or only transitionally express basal cytokeratins. P63 is located on chromosome 3q27, belongs to the p53 gene family and plays a crucial role in maintenance of stem cell populations [12-15]. Whereas p63 protein is a specific myoepithelial cell marker in normal breast tissue, it is overexpressed in a subset of highly aggressive breast cancers that represent a basal/myoepithelial phenotype [16].

Although basal-like breast cancers have been characterized for antigen expression, there are limited data about the precursor lesions from which they emerge. To investigate the hypothesis that comedo-ductal carcinoma in situ (DCIS) serves as a potential precursor entity for basal-like breast cancers, we analyzed expression of p63, CK5, Her2, EGFR, ER and PgR in clinical comedo- and noncomedo-DCIS and the MCF10DCIS.com xenograft model of human comedo-DCIS. Comedo-DCIS accounts for ~10% of all DCIS and differs from other DCIS subtypes in being ER^−^, PgR^−^, Her2/neu+ and having the highest risk for progression and post-operative recurrence [17,18] Comedo-DCIS tumors are easily distinguished from other DCIS by the characteristic central comedo necrosis [19]. Here we provide evidence for a precursor link between comedo-DCIS and basal-like breast cancer, and document the presence of a novel clinical p63+/Her2/neu coexpressing subgroup that serve as progenitors for basal-like breast cancer.

## RESULTS

### Expression of basal markers in comedo-DCIS

**Figure 1:**
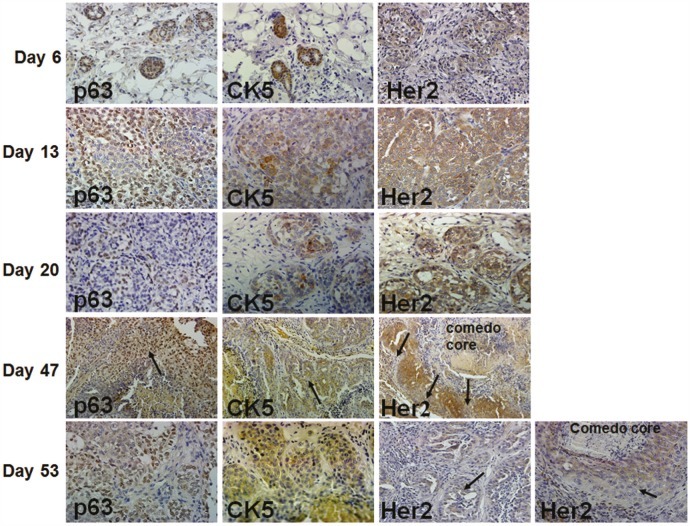
Immunohistochemical analysis of p63, CK5 and Her2/neu in MCF10DCIS.com xenografts In day 47 xenografts, arrows indicate p63 and CK5 staining in infiltrating tumor cells, and decreased Her2/neu expression in cells near the basal surface. Her2/neu expression is reduced in microinvasive (long arrow) and luminal (short arrow) areas of advanced comedo-DCIS xenografts at day 53. The data are representative of at least five independent tumors. Magnification X10 (day 47); X20 (days 6 and 20), and X40 (days 13 and 53).

When implanted into the mammary fatpads of immunodeficient female nude mice, MCF10DCIS.com cells produce solid DCIS lesions that reproducibly progress to comedo-DCIS [20-22]. The kinetics of DCIS development and progression to comedo-DCIS and invasive carcinoma of MCF10DIS.com xenografts, and expression of p63, CK5, Her2/neu and EGFR are shown in Fig. 1. At day 6 of MCF10DCIS.com cell implantation, the cells begin to organize into ducts that express p63, CK5 and Her2/neu. By day 13, the ducts become solid lesions that overexpress Her2/neu. These lesions also show increases in p63 expression whereas CK5 expression is only focally detected, and EGFR expression is negligible (data not shown). ER and PgR expressions are undetectable (data not shown). At day 20, the solid lesions are classified as DCIS as they contain a myoepithelial layer and basement membrane [20-22]. P63 expression is either restricted to myoepithelial cells or is present in both the myoepithelial and luminal areas of DCIS; however, Her2/neu expression remains widespread. By day 35, the solid DCIS lesions progress to comedo-DCIS that are characterized by the classical central comedo necrotic core [20-22, and data not shown]. By day 47, these lesions progress to advanced comedo-DCIS where there is abundant loss of luminal epithelial cells and loss of the basement membrane. P63 and CK5 expressions are maintained in infiltrating invasive cancer cells whereas Her2/neu expression is reduced in invasive and basally located cancer cells (Fig. 1, arrows in Her2 staining in Days 47 and 53 lesions). These data suggest that comedo-DCIS lesions derived from MCF10DCIS.com cells contain progenitor cells that are capable of giving rise to typical basal-like breast cancer.

### MCF10DCIS.com cells selected for high CK5 expression produce Her2/neu positive lesions that overexpress p63 and CK5

**Figure 2:**
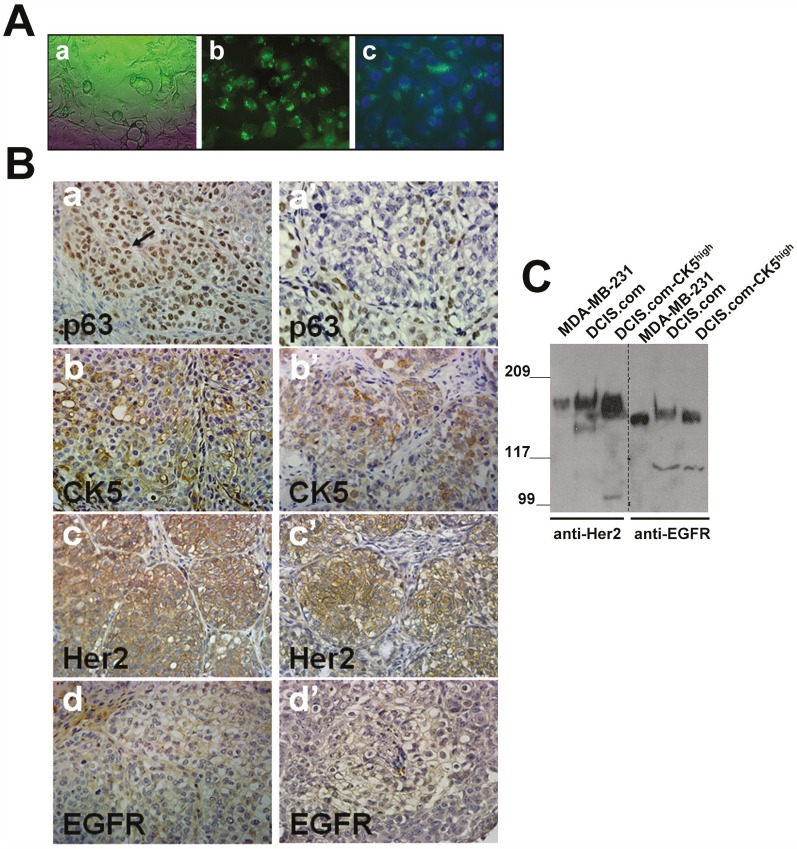
Her2/neu is overexpressed in MCF10DCIS.com subpopulations selected for CK5 overexpression A. MCF10DCIS.com cells expressing CK5 promoter directed ZsGreen reporter expression. A, phase contrast micrograph; b and c, MCF10DCIS.com-CK5^high^ cells isolated by FACS; c, DAPI stained MCF10DCIS.com-CK5^high^ cells. Magnification X20. B. Comparison of p63, CK5, Her2/neu and EGFR staining in day 20 xenografts derived from MCF10DCIS.com-CK5^high^ (a-d) and unselected parental MCF10DCIS.com (a'-d') cells. Data are representative of five independent tumors. C. Western blot analysis of Her2/neu and EGFR in MDA-MB-231, MCF10DCIS.com and MCF10DCIS.com-CK5^high^ whole cell lysates.

To determine the relationship between expression of basal markers p63/CK5 and luminal marker Her2/neu in the pathogenesis of basal-like breast cancer, MCF10DCIS.com subpopulations overexpressing CK5 were isolated from MCF10DCIS.com cells using a ZsGreen1 reporter plasmid [23] in which expression of the reporter is directed by the CK5 promoter (Fig. 2A). MCF10DCIS.com-CK5^high^ or unfractionated MCF10DCIS.com cells were implanted into the inguinal mammary fatpads, and lesions harvested at day 20 were compared for expression of p63, CK5, EGFR and Her2/neu. As shown in Figure 2B, whereas unfractionated MCF10DCIS.com cells organize into classical DCIS with p63 expression either restricted to the myoepithelial layer or present in both the myoepithelial and luminal compartments, lesions produced by MCF10DCIS.com-CK5^high^ cells showed overexpression of p63 positive cells in both the myoepithelial and luminal regions (Fig. 2B, compare panels a with a'). Consistent with the high CK5 promoter activity of MCF10DCIS.com-CK5^high^ cells, the tumors derived from these cells overexpress CK5 (Fig. 2B, compare b with b'). However, as with tumors derived from unfractionated MCF10DCIS.com cells, CK5 expression was observed in both luminal and basally located cells of the in situ lesions. These data suggest that the CK5+ subpopulation isolated from MCF10DCIS.com cells are adult progenitor cells which can differentiate into myoepithelial and luminal cells in vivo. Basal cytokeratin (CK5/CK14/CK17) reactive tumors have been strongly correlated with high EGFR expression and inversely with Her2/neu and ER/PgR expression [24]. Interestingly, EGFR expression was weak or negligible in the tumors derived from both MCF10DCIS.com-CK5^high^ and unfractionated MCF10DCIS.com cells (Fig. 2B, panels d and d') whereas Her2/neu was strongly expressed in both (Fig. 2B, compare c with c'). To verify whether the EGFR and Her2/neu expression pattern in the tumors reflected those in the cell lines used for xenografts assays, total cell lysates were prepared from MCF10DCIS.com, MCF10DCIS.com-CK5^high^ and MDA-MB-231 (positive control) breast cancer cells and analyzed for Her2/neu and EGFR protein expression by immunoblotting with the EGFR and Her2/neu antibodies used for immunohistochemical staining. As shown in Fig. 2C, Her2/neu and EGFR proteins are robustly expressed in both MCF10DCIS.com and MCF10DCIS.com-CK5^high^ cell lines. These data validate the immunoreactivity of the EGFR antibody, and confirm that EGFR expression is lost in vivo whereas Her2/neu expression is maintained. These results potentially reveal a novel CK5/Her2/neu expressing subgroup in comedo-DCIS that is distinct from the reported CK5/EGFR subgroup [24].

### P63/Her2/neu coexpression in comedo-DCIS

**Figure 3:**
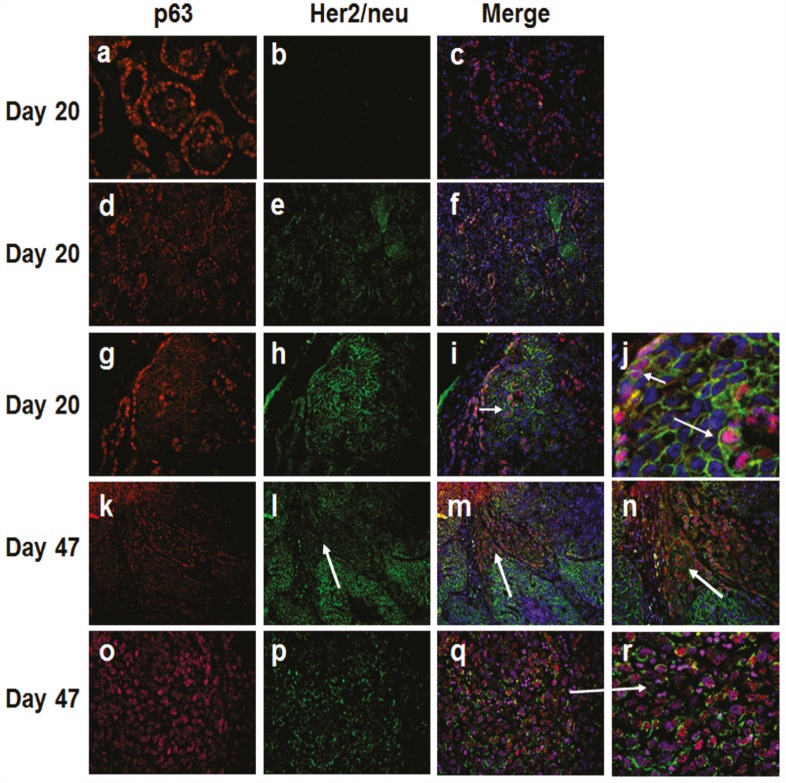
Immunofluorescence analysis of p63 and Her2/neu in MCF10DCIS.com xenografts Note the presence of in situ carcinomas with p63 expression restricted to the myoepithelium and lacking Her2/neu (a-c), and those with p63 and Her2/neu expressions (d-f and g-j). Short arrow in panel j shows p63 and Her2/neu coexpression in the myoepithelial cells. The long arrow in panel j and the arrow in panel i show p63 and Her2/neu expression in the luminal region of DCIS. Panel j is a higher magnification of panel i. Panels k-n, expansion of infiltrating p63 positive cancer cells with decreased Her2/neu expression (arrows in l-n). Panel n is higher magnification of panel m. Panels o-r, infiltrating cancer cells coexpressing p63 and Her2/neu. Panel r is a magnified image of panel q (arrow). The results are representative of three independent experiments. Magnifications X20, panels d-f, k-m; X40, a-c, g-i, o-q; X100, n; X200, j and r.

To further verify the presence of a basal cytokeratin/Her2/neu expressing subgroup and its relevance to emergence of basal-like breast cancer, we performed immunofluorescence double labeling with digital image processing of P63 (a bonafide basal and myoepithelial marker) and Her2/neu in day 20 and day 47 MCF10DCIS.com xenografts, normal breast tissues, and clinical comedo-DCIS patient samples. Immunofluorescence analysis of day 20 MCF10DCIS.com lesions consistently revealed two patterns of p63 and Her2/neu expression: (i) ducts in which p63 expression is restricted to the myoepithelial layer and no Her2/neu expression (p63+/Her2/neu^−^; Fig. 3, panels a-c), and (ii) ducts with cells coexpressing p63 and Her2/neu (p63+/Her2/neu+; Fig. 3, panels d-f and g-i). In the latter, p63 and Her2/neu coexpression is seen in both the basally situated cells in contact with the basement membrane (Fig. 3, panels g-i and short arrow in panel j) and luminal cells located in the interior of the ducts (Fig. 3, panels g-i and long arrow in panel j). In advanced comedo-DCIS tumors (day 47 MCF10DCIS.com xenografts), invasive cancer cells show either strong p63 and decreased/weak Her2/neu coexpression (Fig. 3, panels k-n, arrows in panels l-n), or continued coexpression of p63 and Her2/neu (Fig. 3, panels o-r).

**Figure 4:**
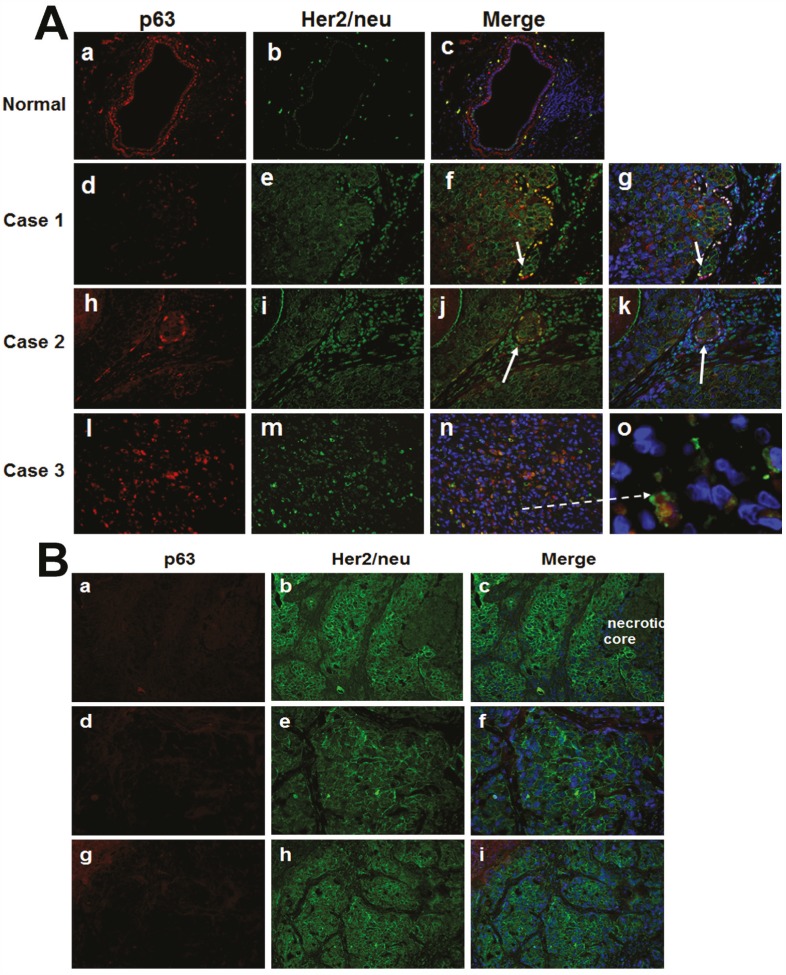
Immunofluorescence analysis of p63 and Her2/neu in clinical comedo-DCIS (A) and SUM-225 xenografts (B) A. Panels a-c, staining in normal ducts; panels d-o, clinical cases of comedo-DCIS. Arrows show microinvasive cancer cells with p63 and Her2/neu expression. Panel o is a magnified image of panel n (dashed arrow), and shows p63/Her2/neu coexpressing infiltrating tumor cells. B. a-c, early SUM225 lesions resembling comedo-DCIS; d-f and g-i, ductal infiltrating carcinomas in advanced SUM225 tumors. Note strong Her2/neu staining and absence of p63 expression in SUM225 tumors; the results are representative of five independent experiments. Magnifications X400 (panel o in A), all others X20.

Immunofluorescence double labeling of p63 and Her2/neu in clinical comedo-DCIS breast tumors showed similar data as those seen in MCF10DCIS.com tumors. In normal ducts, Her2/neu staining is negligible and p63 is exclusively localized to the myoepithelial cells (Fig. 4A, panels a-c), whereas microinvasive cancer cells adjacent to comedo-DCIS show positivity for both p63 and Her2/neu (Fig. 4A, panels f, g, j and k) or coexpression of p63/Her2/neu in malignant tumor cells (Fig. 4A, arrow in panel o). These data resemble the results from MCF10DCIS.com xenografts and provide clinical support for the presence of the novel subgroup coexpressing basal markers and Her2/neu, and for a novel link between comedo-DCIS and basal-like breast cancer.

To determine whether the p63/Her2/neu expressing cells represent a subgroup that is unique to comedo-DCIS, we performed similar immunofluorescence double labeling analysis of p63 and Her2/neu in tumors generated from SUM225 human breast cancer cells. SUM225 cells overexpress Her2/neu [25,26], and produce infiltrating ductal carcinomas in vivo that morphologically mimic comedo-DCIS in that they have central necrosis but lack collagen IV-reactive basement membrane that is characteristic of DCIS (data not shown; manuscript in preparation). SUM-225 tumors show strong Her2/neu expression and are negative for p63 in early lesions resembling comedo-DCIS (Fig. 4B, a-c) as well as in advanced tumors with infiltrating ductal carcinoma (Fig. 4B, d-f and g-i). These data suggest that the p63/Her2/neu coexpressing cells represent a subgroup that is unique to comedo-DCIS, and that the classic Her2-overexpressors and atypical basal-like p63+/Her2+ cells potentially emerge from distinct precursor subsets.

### P63 and Her2/neu expression are associated with clinical comedo-DCIS recurrence

**Figure 5:**
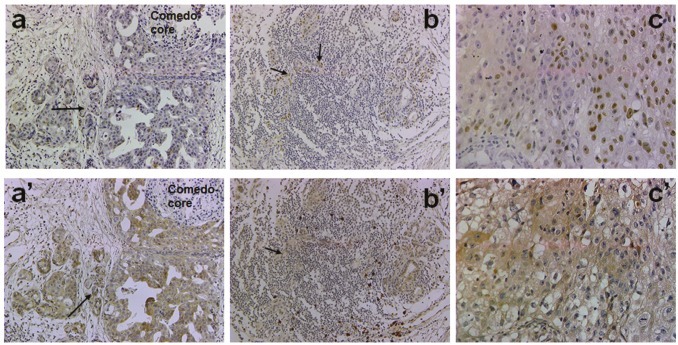
Immunohistochemical analysis of p63 and Her2/neu in clinical comedo-DCIS with recurrence Panels a-c, p63 and a'-c', Her2/neu. Panels a, a': staining in microinvasive cancer cells (arrows); b, b': cancer cell staining in lymphocytic infiltrates (arrows); c, c': staining in invasive cancer cells. Magnification X20 (a, a', b, b'); X40 (c, c').

The data in Table 1 show results of p63 and Her2/neu immunohistochemical analysis in clinical comedo-and noncomedo-DCIS tumors that recurred or showed microinvasion. The occurrence of p63 expression in invasive cancer cells was significantly associated with comedo-type DCIS, and was positive in 88% of the cases (*P* = 0.038). Her2/neu positivity did not show a similar association as moderate to strong Her2/neu staining was observed in tumors of both comedo- and noncomedo-DCIS types (*P* = 0.669). Simultaneous expression of p63 and Her2/neu was marginally associated with comedo-DCIS (*P* = 0.067). Figure 5 shows a typical staining for p63 and Her2/neu in microinvasive (arrows in a, a'), infiltrating (arrows in b and b'), and malignant (c and c') areas of clinical comedo-DCIS.

**Table 1:** Immunohistochemical analysis of p63 and Her2/neu in clinical DCIS

Comedo-DCIS		Noncomedo-DCIS	
p63	Her2/neu	p63	Her2/neu
+ (15/17)*	+ (14/17)	+ (6/12)	+ (9/12)

p63 +, staining in infiltrating tumor cells; Her2/neu +, moderate to strong staining in infiltrating tumor cells. *, indicates p63 association with comedo-DCIS microinvasion/recurrence P = 0.038.

## DISCUSSION

**Figure 6:**
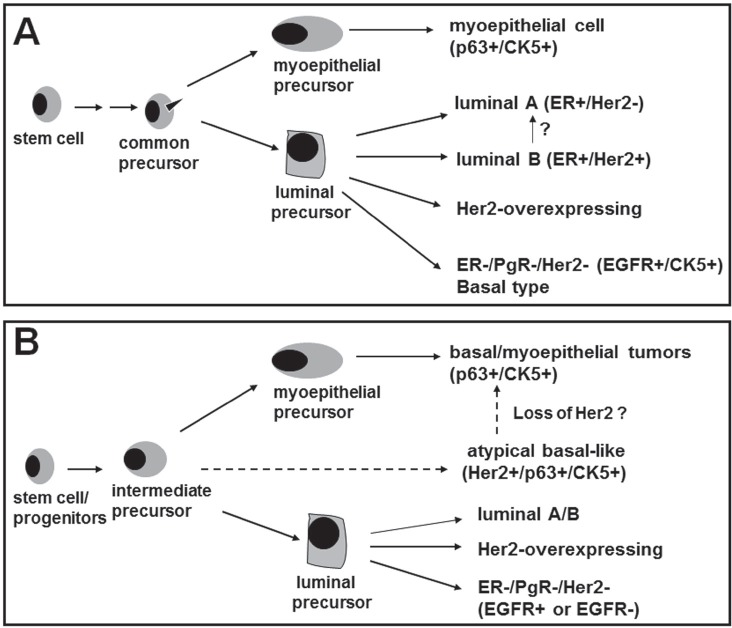
A novel model for emergence of basal-like breast cancer and breast cancer heterogeneity Model A, the documented model for emergence of the known subtypes of breast cancer. Model B shows the identification of a novel subgroup that expresses p63, CK5 and Her2/neu while lacking EGFR expression. Our data suggest that comedo-DCIS contains this novel subgroup that serve as precursors for basal-like breast cancers. The p63/Her2/neu coexpressing tumor cells detected in comedo-DCIS could either give rise to p63+/Her2^−^ tumors (typical basal-like triple negative cancer) or p63+/Her2+ tumors (atypical basal-like cancer). P63/Her2 coexpressing cancer cells probably represent an intermediate or transitional progeny and its accumulation suggest a potential block in the differentiation process.

In this paper, we have identified for the first time a novel p63/Her2/neu coexpressing subgroup that provides a precursor link between comedo-DCIS and basal-like breast cancer. Based on our results, we have proposed a model in Fig. 6 to explain the mechanisms by which basal-like tumors could arise from comedo-DCIS. The Model A in Fig. 6 shows the previously recognized pathways for generation of basal, luminal A, luminal B and classic Her2-overexpressing breast cancers. Based on our data that p63 and Her2/neu are coexpressed in clinical comedo-DCIS and the MCF10DCIS.com comedo-DCIS model, we suggest that the p63 and Her2/neu expressors share a common precursor. In Model B (Fig. 6), we propose that p63+/Her2+ breast cancer cells represent an intermediate progeny of stem cell differentiation. That p63+/Her2+ cells are detected both in the myoepithelial and luminal compartments of comedo-DCIS suggests that these transitional precursors probably experience a block in differentiation into discrete p63+/Her2/neu^−^ (basal cells of myoepithelial lineage) and Her2+/p63^−^ (Her2-overexpressing) lineages. However, since no p63 expression is detected in Her2/neu overexpressing SUM225 tumors, it is not clear if the classic Her2/neu overexpressing lineage is derived from a separate precursor or from p63+/Her2+ precursors that have a different differentiation program. Since SUM225 cells produce infiltrating ductal carcinomas despite their morphologic resemblance to comedo-DCIS [27], it is tempting to speculate that the p63+/Her2+ coexpressing subset may be unique to DCIS of the comedo subtype. CK5 immunoreactive tumors are generally negative for ER and Her2/neu but show strong correlation with increased EGFR expression [24]. Interestingly, EGFR expression is weak or undetectable in MCF10DCIS.com xenografts, suggesting that EGFR is not obligatory for the pathogenesis of this basal-like breast cancer subtype.

Myoepithelial and luminal epithelial cells have different immunoprofiles [28]. Myoepithelial cells of in situ lesions express p63 and basal cytokeratins CK5 and CK17 that are useful for distinguishing benign from malignant lesions [6,7,29,30]. Our data from MCF10DCIS.com-CK5^high^ tumors show that despite selection of MCF10DCIS.com subpopulations with high CK5 expression, the tumors produced from these cells strongly express CK5 in both luminal and basal cells in the duct, and resemble those derived from unfractionated MCF10DCIS.com cells. By immunofluorescence double labeling experiments, Böcker et al [31] demonstrated that CK5+ cells are adult progenitor cells which can differentiate to glandular epithelium or myoepithelial cells by passing through either CK5/CK8/CK18/CK19 or CK5/α-smooth muscle antigen positive intermediates. That the CK5+ enriched fraction of MCF10DCIS.com cells possesses progenitor property is supported by strong and comparable expression levels of the luminal protein Her2/neu in tumors derived from unfractionated MCF10DCIS.com cells. p63 is a classic myoepithelial marker that outlines the myoepithelium of the normal breast; however, expansion of p63+ cells in basal-like breast cancer occurs from differentiation of precursors that give rise to the myoepithelial lineage or from precursors that are scattered at the basal and luminal areas of the ducts. We posit that both these scenarios take place as our data from MCF10DCIS.com derived lesions show two distinct types of ducts: one in which p63 is restricted to the myoepithelium and negative for Her2/neu, and the other in which p63 and Her2 coexpression is seen in both basal and luminal compartments. Studies on fetal and infant breasts have shown that cells at the tips of the lobular buds and terminal end buds have a characteristic cytoskeletal protein profile that is associated with their capacities to generate both basal and luminal type cells [32].

Our data show that Her2/neu expression is decreased or lost in invasive cancer cells of some comedo-DCIS lesions. In such an event, proliferation of these cells could give rise to the typical basal-like triple negative breast cancer. However, our data from the MCF10DCIS.com model also show preservation of p63/Her2 coexpression in the invasive areas of comedo-DCIS lesions. In this case, expansion of these cells could give rise to atypical p63/Her2-expressing basal-like carcinoma. In such an event, coexpression of p63 and Her2 could potentially direct novel or modified gene expression programs that could further contribute to tumor heterogeneity. Regardless of whether the intermediate p63+/Her2+ precursors differentiate into p63+/Her2^−^ or remain p63+/Her2+, Her2 activity may play a contributory role in the pathogenesis of basal-like breast cancers originating from comedo-DCIS. Her2/neu targeted therapy may be beneficial to patients with p63/Her2 coexpressing comedo-DCIS. On the flip side, targeting Her2/neu could potentially enhance expansion of p63+/Her2^−^ progeny and consequently promote transition to typical basal-like breast cancer.

DCIS is a ‘heterogenous’ collection of lesions with diverse malignant potential. Among the various subtypes of DCIS, comedo-DCIS is associated with high nuclear grade, aneuploidy [33], higher proliferation rates [34], and *Her2/neu* gene amplification or protein overexpression [35,36], ER negativity, and clinically aggressive behavior [37,38]. These cells also have a higher probability of developing malignant ductal carcinoma. The presence of DCIS in particular of the comedo-type with invasive basal-like carcinomas has been reported [39]. These clinical in situ lesions displayed the same immunophenotype (positive for CK5/6, CK14, CK17 and EGFR, and negative for Her2/neu and ER/PgR) as the invasive basal-like cancer component, suggesting a common precursor lesion [39]. While our data provide strong evidence for a common link between comedo-DCIS and basal-like cancer, our results differ from those of Dabbs *et al* [39] in that a novel p63/CK5/Her2/neu expressing subgroup play an important role in this link.

Based on our data we propose that similar to “DCIS”, triple negative breast cancer is not a single disease that is identifiable by triple negativity for ER/PgR/Her2/neu [6-11], and positivity for EGFR and CK5 expression, but rather are dynamic entities that may be generated in varying proportions or as transitional intermediates in the heterogeneous milieu of the breast tumor. The relationship between comedo-DCIS and basal-like breast cancer illustrates this point, and suggests that p63/Her2/neu expressions may be used as markers for identifying breast cancers that may progress to a novel clinical subgroup of basal-like breast cancer.

## MATERIALS AND METHODS

### Cell lines and culture

MCF10DCIS.com human breast cancer cells were maintained in DMEM/F12 medium (1:1) supplemented with 5% horse serum and 4 mM glutamine [20-22]. Cells were maintained in a 37°C humidified incubator with an atmosphere of 5% CO_2_. To isolate MCF10DCIS.com subpopulations overexpressing CK5, MCF10DCIS.com cells were transfected with a pZsGreen1 reporter plasmid in which expression of the ZsGreen1 reporter is placed under the control of the CK5 promoter. Cells were sorted in BD FACSDiVa, and the top 10% of cells with the highest ZsGreen fluorescence (referred as MCF10DCIS.com-CK5^high^) were collected into 50% fetal bovine serum in phosphate buffered saline as described previously [23] and propagated. SUM225 human breast cancer cells were maintained in DMEM/F12 medium supplemented with 5% fetal bovine serum, 5 μg/ml insulin and 1 μg/ml hydrocortisone [40]. MDA-MB-231 cells were maintained in DMEM/F12 medium supplemented with 5% fetal bovine serum [41].

### Generation of MCF10DCIS.com and SUM-225 xenografts

Xenograft tumors were generated by injecting 2 × 10^6^ (MCF10DCIS.com or MCF10DCIS.com-CK5^high^) or 5 × 10^6^ SUM-225 cells, respectively, in 0.1 ml Matrigel subcutaneously into the inguinal fatpads of mammary glands #4 and #9 of 6-8 female nude mice per group [20-22]. MCF10DCIS.com xenografts were removed at various periods of growth, and SUM-225 xenografts were harvested at 60-90 days postimplantation. Tumor tissues were fixed in 10% buffered formalin. In vivo experiments were approved by the Institutional Animal Care and Use Committee, and conformed to the NIH regulatory standards.

### Clinical DCIS breast tumors

Anonymized DCIS breast tumor sections from 17 comedo-DCIS with microinvasion or recurrence and 12 noncomedo-DCIS with microinvasion or recurrence were acquired after protocol review and approval by the Wayne State University Human Investigation Committee.

### Immunohistochemistry and immunofluorescence

Immunohistochemistry and immunofluorescence double labeling were performed on paraffin-embedded xenografts and clinical comedo- and noncomedo-DCIS as previously described [22,23]. For immunohistochemical analysis, proteins were detected with appropriate biotinylated secondary antibodies and HRP-conjugated streptavidin. Nuclei were counterstained with hematoxylin. For immunofluorescence double lebeling, proteins were detected with FITC- or Texas Red-conjugated secondary antibodies, and counterstained with 4'6-diamidino-2-phenylindole (DAPI). Slides were stained in the absence of primary antibody or with isotype-matched nonimmune IgG to assess nonspecific reactions. Images were collected on Olympus BX60 microscope equipped with Sony high resolution/sensitivity CCD video camera and digitally processed with CellSens imaging software. The antibodies used for immunohistochemistry and/or immunofluorescence were: anti-p63 (Santa Cruz), anti-CK5 (Dako), anti-Her2 (clone CB11, Life Technologies), anti-EGFR (Cell Signaling), anti-type IV collagen (Dako), anti-ER and anti-PgR (Dako). Immunohistochemistry slides were semi-quantitated as follows: P63 staining was scored as - (negative) or + (strong nuclear staining); CK5 was graded as - (negative) or + (≥ 10% cells positive); EGFR and Her2 were graded as - (negative), + (moderate) and ++ (strong) membrane staining in at least 10% of tumor cells. ER and PgR receptors were scored as - (negative) or + for nuclear immunoreactivities to the hormone receptors.

### Western blot analysis

Whole cell lysates were prepared from MCF10DCIS.com, MCF10DCIS.com-CK5^high^ or MDA-MB-231 monolayers as described previously [22,41] and aliquots of lysates containing 25 μg of protein were subjected to SDS-PAGE and Western blot analysis of Her2 (clone CB11, Life Technologies) and EGFR (Cell Signaling).

### Statistical analysis

The associations between expression of p63 or Her2/neu singly and in combination with comedo- vs. noncomedo-DCIS histological types were tested using the 2-sided Fisher's exact test. A *P* value <0.05 was considered significant.
